# Mitochondria: the hidden engines of traumatic brain injury-driven neurodegeneration

**DOI:** 10.3389/fncel.2025.1570596

**Published:** 2025-05-09

**Authors:** Olusola A. Olatona, Sydney P. Sterben, Sahan B. S. Kansakar, Aviva J. Symes, Volha Liaudanskaya

**Affiliations:** ^1^Department of Biomedical Engineering, University of Cincinnati, Cincinnati, OH, United States; ^2^Department of Pharmacology and Molecular Therapeutics, Uniformed Services University, Bethesda, MD, United States; ^3^Neuroscience Graduate Program, University of Cincinnati, College of Medicine, Cincinnati, OH, United States

**Keywords:** brain injury, mitochondria, neurodegeneration, metabolism, bioenergetics

## Abstract

Mitochondria play a critical role in brain energy metabolism, cellular signaling, and homeostasis, making their dysfunction a key driver of secondary injury progression in traumatic brain injury (TBI). This review explores the relationship between mitochondrial bioenergetics, metabolism, oxidative stress, and neuroinflammation in the post-TBI brain. Mitochondrial dysfunction disrupts adenosine triphosphate (ATP) production, exacerbates calcium dysregulation, and generates reactive oxygen species, triggering a cascade of neuronal damage and neurodegenerative processes. Moreover, damaged mitochondria release damage-associated molecular patterns (DAMPs) such as mitochondrial DNA (mtDNA), Cytochrome C, and ATP, triggering inflammatory pathways that amplify tissue injury. We discuss the metabolic shifts that occur post-TBI, including the transition from oxidative phosphorylation to glycolysis and the consequences of metabolic inflexibility. Potential therapeutic interventions targeting mitochondrial dynamics, bioenergetic support, and inflammation modulation are explored, highlighting emerging strategies such as mitochondrial-targeted antioxidants, metabolic substrate supplementation, and pharmacological regulators of mitochondrial permeability transition pores. Understanding these mechanisms is crucial for developing novel therapeutic approaches to mitigate neurodegeneration and enhance recovery following brain trauma.

## 1 Mitochondria structure and function

The adult human brain accounts for about 2% of total body weight but consumes approximately 20% of the body’s energy supply to sustain its high metabolic activity. The energy demand is even higher in young and developing brains; a newborn consumes about 60% of the body’s daily energy, while a 10 years-old child’s brain uses around 50% of the body’s total basal metabolic rate (Goyal et al., 2014; Steiner, 2019). Neurons account for 75%–80% of the brain’s energy consumption (Belenguer et al., 2019; Chakrabarty and Chandel, 2021; Chen W. et al., 2023; Collier et al., 2023) and rely heavily on mitochondria, the primary producers of cellular energy, generating up to 95% of a eukaryotic cell’s ATP to support essential functions such as action potential generation, ion homeostasis, neurotransmitter cycling, and synaptic remodeling for learning and memory.

**FIGURE 1:**
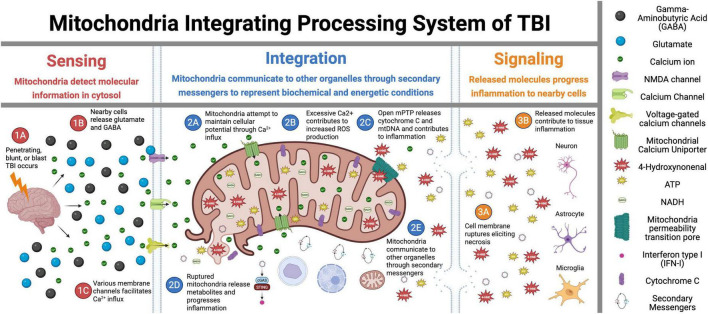
Mitochondrial signal transduction in traumatic brain injury (TBI). This figure illustrates the sequential role of mitochondria in TBI across three critical phases: Sensing, Integration, and Signaling. In the Sensing phase **(1A–1C)**, the initial trauma triggers glutamate release and activation of membrane ion channels, initiating the early cellular responses. During the Integration phase **(2A–2E)**, mitochondria interpret injury signals through calcium influx, reactive oxygen species (ROS) production, and mitochondria permeability pore (mPTP) opening while also interacting with other cellular components. The Signaling phase **(3A–3E)** demonstrates how these integrated responses lead to mitochondria membrane rupture, necrosis, and inflammatory propagation to surrounding tissues. Overall, this figure depicts the central role of mitochondria as information processors that detect initial injury signals, integrate biochemical responses, and ultimately determine cellular fate in the progressive pathophysiology of TBI. Figure created in Biorender.com.

Over the last decade, it has become appreciated that mitochondria are better understood as “cell processors” rather than simply energy-generating “powerhouses” (Picard and Shirihai, 2022). Numerous studies have demonstrated mitochondria as dynamic organelles that transform energy, synthesize biomolecules, and act as critical signaling hubs to transduce and integrate biological information (Bader and Winklhofer, 2020; Lamade et al., 2020; Norat et al., 2020; Picard and Shirihai, 2022). In this role, in coordination with the nucleus and other organelles, mitochondria form the mitochondrial information processing system (Picard and Shirihai, 2022). This system is composed of three steps (Figure 1): mitochondria (1) sense internal and environmental stimuli through changes in morphology and function; (2) integrate information via dynamic, network-based physical interactions and diffusion mechanisms; and (3) regulate the functions of other organelles and systemically modulate physiology. This complex multistep system enables mitochondria to transduce metabolic, biochemical, neuroendocrine, and other local or systemic signals that enhance the organism’s adaptability. As a result, mitochondria have been recognized as critical regulators of ATP production, inflammation, death progression, metabolism, and epigenetic state.

Mitochondria are double membrane organelles, with each membrane playing distinct and crucial roles (Giorgi et al., 2015). The outer membrane (OMM) is a “bodyguard,” containing porins that regulate the ions, nutrients, and small molecules (up to about 5 kDa) flowing into the intermembrane space. This permeability is essential for exchanging metabolites and ions between the mitochondria and the cytosol. Additionally, the OMM plays a significant role in host defense and regulation of apoptosis. It houses proteins such as B-cell lymphoma 2 (Bcl-2) family members that control Cytochrome C release from the intermembrane space into the cytosol, triggering the intrinsic apoptosis pathway [for a detailed review, see Desagher and Martinou (2000)]. Furthermore, the OMM engages in lipid synthesis and the import of lipids from the endoplasmic reticulum, facilitating lipid exchange with other organelles (Crompton, 1999; Giorgi et al., 2015; Giacomello et al., 2020). Finally, the OMM initiates, regulates, and executes mitochondrial fission, fusion, and mitophagy, critical components of mitochondrial homeostasis (Liesa and Shirihai, 2013; Di Pietro et al., 2017; Herst et al., 2017; Misgeld and Schwarz, 2017; Giacomello et al., 2020; Chen W. et al., 2023).

The inner mitochondrial membrane (IMM), tightly packed into the membrane cristae, is the site of oxidative phosphorylation (OXPHOS), the primary ATP production machinery. The IMM contains the electron transport chain (ETC) and ATP synthase protein complexes, which conduct this process (see chapter “2.1 Brain bioenergetics” for more details) (Kühlbrandt, 2015; Audano et al., 2020; Giacomello et al., 2020). Unlike the OMM, the IMM is impermeable to most ions and molecules, a feature critical for maintaining the proton gradient established by the ETC. This proton gradient drives ATP synthesis through ATP synthase (Paumard et al., 2002). Additionally, the IMM is equipped with numerous transport systems that regulate the movement of metabolites across the membrane, ensuring the efficient production and distribution of energy within the cell (Picard and Shirihai, 2022).

## 2 Mitochondria dysfunction as primary damage post-traumatic brain injury

Mitochondrial dysfunction refers to mitochondria failing to perform one of their critical functions: sensing, integration, or signaling (Picard and Shirihai, 2022). When mitochondrial function is compromised, altered bioenergetic and metabolic processes can lead to increased oxidative stress, inflammation and/or cell death. Mitochondrial dysfunction has many potential causes, including genetic mutations, oxidative stress, environmental toxins, aging, or trauma. Mitochondrial dysfunction and defective mitochondrial dynamics are proposed as key mechanisms with functional importance in the early stages of Alzheimer’s disease, Parkinson’s disease, Amyotrophic lateral sclerosis, and other neurodegenerative diseases, as well as after TBI (Lin and Beal, 2006; Nunnari and Suomalainen, 2012; Nicolson, 2014; Hiebert et al., 2015; McGovern and Barreto, 2021; Picard and Shirihai, 2022; Strope et al., 2022; Schmitt et al., 2023). Specifically, TBI (Bader and Winklhofer, 2020) disrupts key processes such as energy production, metabolic regulation, calcium homeostasis, and oxidative stress management. Damage to the electron transport chain (ETC) impairs oxidative phosphorylation (OXPHOS), leading to ATP depletion and bioenergetic failure (Hiebert et al., 2015; Kilbaugh et al., 2015; Herst et al., 2017; Hubbard et al., 2018; Leipnitz et al., 2018; Lyons et al., 2018; Belenguer et al., 2019; Hubbard et al., 2019; Ahluwalia et al., 2021; Pandya et al., 2021; Bhatti et al., 2022; Cheng et al., 2022; Strope et al., 2022; Hubbard et al., 2023), while disruptions in mitochondrial dynamics hinder neuronal repair and survival (Yonutas et al., 2016; Lyons et al., 2018; Hubbard et al., 2019; Lazzarino et al., 2019; Ahluwalia et al., 2021). Excessive oxidative stress (Tavazzi et al., 2005; Shi and Gibson, 2007; Hiebert et al., 2015; Hubbard et al., 2018), excitotoxicity, and metabolic dysregulation (Arun et al., 2013; Yonutas et al., 2016; Hubbard et al., 2018; Lyons et al., 2018; Hubbard et al., 2019) further exacerbate neuronal damage, contributing to neurodegeneration and prolonged functional deficits (Choi et al., 2004; Clark et al., 2006; Lin and Beal, 2006; Shi and Gibson, 2007; Trushina and McMurray, 2007; Keating, 2008; Bhat et al., 2015; Bhatti et al., 2017; Rana et al., 2020; Ahluwalia et al., 2021; Fesharaki-Zadeh, 2022). In the next two chapters, we will explore these mitochondrial impairments in the context of brain trauma, highlighting their impact on neuronal survival and potential therapeutic targets.

### 2.1 Brain bioenergetics

Brain cells generate energy through four main mechanisms: glycolysis, the tricarboxylic acid (TCA) cycle, OXPHOS, and fatty acid oxidation (Belenguer et al., 2019; Benaroya, 2020). Neuronal cells rely on OXPHOS, and proliferating glial cells predominantly utilize glycolysis for ATP production.

**FIGURE 2:**
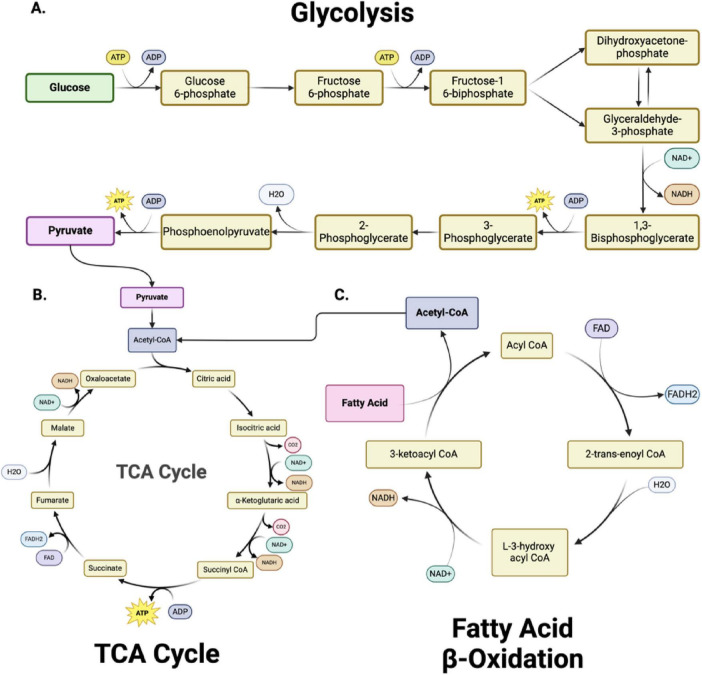
Metabolic pathways and bioenergetics. **(A)** Glycolysis, **(B)** The Tricarboxylic acid cycle (TCA) cycle and **(C)** Fatty Acid β-Oxidation are metabolically interconnected, with key intermediates serving as inputs for downstream pathways. In glycolysis **(A)**, glucose is converted into pyruvate through 10 enzymatically controlled steps, producing adenosine triphosphate (ATP) and reduced nicotinamide adenine dinucleotide (NADH). Pyruvate subsequently enters the TCA cycle **(B)** as acetyl-CoA, driving the production of NADH and FADH_2_ (reduced flavin adenine dinucleotide) for oxidative phosphorylation. Fatty acid β-oxidation **(C)** degrades fatty acids into acetyl-CoA, further fueling the TCA cycle and linking lipid metabolism to energy production. Together, these pathways sustain cellular bioenergetics by integrating carbohydrate and lipid metabolism. Figure created in Biorender.com.

Glycolysis - is a fundamental metabolic pathway crucial in cellular energy production (Figure 2A). It involves the breakdown of glucose, a six-carbon sugar, into two pyruvate molecules, each containing three carbons (Goyal et al., 2014; Carpenter et al., 2015; Steiner, 2019; Devanney et al., 2020; Xu et al., 2021; Wei et al., 2023). This cytoplasmic process is the first step in both aerobic and anaerobic respiration. The glycolytic pathway begins with the phosphorylation of glucose to form glucose-6-phosphate, a reaction catalyzed by the enzyme hexokinase (Liu et al., 2023). This initial step requires ATP, which invests energy to activate glucose for further breakdown. The pathway proceeds through a series of enzymatic steps, ultimately splitting the six-carbon molecule into two three-carbon molecules of glyceraldehyde-3-phosphate. Each glyceraldehyde-3-phosphate molecule then undergoes further transformations, resulting in the production of pyruvate.

Throughout glycolysis, several key reactions generate energy-rich molecules. Specifically, two molecules of NAD^+^ (nicotinamide adenine dinucleotide, oxidized) are reduced to NADH, capturing high-energy electrons. Additionally, substrate-level phosphorylation occurs twice, producing four ATP molecules. Since two ATP molecules are consumed in the initial steps, the net gain from glycolysis is two ATP molecules per glucose molecule. This modest ATP yield is critical for cells, particularly under anaerobic conditions where oxygen is absent and oxidative phosphorylation cannot occur.

The fate of the pyruvate produced in glycolysis depends on oxygen availability. Under aerobic conditions, pyruvate is transported into the mitochondria, where it is converted into acetyl-CoA by the enzyme pyruvate dehydrogenase. Acetyl-CoA then enters the TCA cycle (Figure 2B), producing additional reduced nicotinamide adenine dinucleotide (NADH) and (reduced flavin adenine dinucleotide) (FADH_2_) molecules. These molecules donate electrons to the electron transport chain, synthesizing significant ATP through oxidative phosphorylation. This pathway underscores the importance of glycolysis as a precursor to the more efficient aerobic respiration process. In anaerobic conditions, such as in muscle cells during intense exercise, pyruvate is instead converted into lactate by the enzyme lactate dehydrogenase (Schurr and Rigor, 1998). This conversion regenerates NAD^+^, allowing glycolysis to continue and produce ATP without oxygen. This anaerobic pathway, known as lactic acid fermentation, provides a rapid but less efficient means of ATP production.

The mitochondrial tricarboxylic acid (TCA) cycle (also called Krebs or citric acid cycle) is essential for energy production in all organs, including the brain. This cycle oxidizes acetyl-CoA into carbon dioxide and high-energy molecules, including ATP, NADH, and FADH_2_. In the first step (Figure 2B), acetyl-CoA combines with oxaloacetate to form citrate (Martínez-Reyes and Chandel, 2020; Abdullah et al., 2022). Through a series of enzyme-catalyzed reactions, citrate is converted back to oxaloacetate, generating various crucial intermediate metabolites (alpha-ketoglutarate, succinyl CoA, succinate, fumarate, malate) and energy-rich molecules (NADH and FADH_2_) in the process. The TCA cycle also produces intermediates used in other biosynthetic pathways, highlighting its central role in cellular metabolism (Nelson et al., 2018) (see section “2.2 Mitochondrial dysfunction and cellular metabolism” for metabolic details).

Oxidative phosphorylation **-** The primary energy-producing mechanism in neurons is oxidative phosphorylation (OXPHOS), which occurs in the mitochondria (Harris et al., 2012; Rangaraju et al., 2014; Magistretti and Allaman, 2015). During this process, electrons from reduced cofactors, such as NADH and FADH_2_ - produced during glycolysis, the TCA cycle, and fatty acid oxidation - are transferred through the electron transport chain (ETC). The ETC comprises a series of protein complexes located in the inner mitochondrial membrane that facilitate the transfer of electrons from electron donors like NADH and FADH_2_ to oxygen, the final electron acceptor. As electrons pass through Complexes I, II, III, and IV, protons are pumped from the mitochondrial matrix to the intermembrane space, creating a proton gradient. The proton motive force drives the synthesis of ATP as protons flow back into the matrix through ATP synthase. The ETC’s role in creating a proton gradient is essential for ATP production through oxidative phosphorylation, and its proper function is vital for cellular energy homeostasis (Kühlbrandt, 2015; Neupane et al., 2019). OXPHOS is highly efficient, yielding approximately 30–36 ATP molecules per glucose molecule, compared to two ATP molecules generated by glycolysis alone.

Fatty acid oxidation (FAO) – FAO within the mitochondria is vital for maintaining mitochondrial bioenergetic capacity (Figure 2C). Cytoplasmic fatty acids are processed to form acyl-CoA molecules (Xiong, 2018; Rose et al., 2020). The carnitine shuttle system transports the acyl-CoA molecules into the mitochondria through the sequential action of carnitine palmitoyltransferase I (CPT I) located on the outer mitochondrial membrane and carnitine-acylcarnitine translocase on the inner mitochondrial membrane. CPT I converts acyl-CoA to acyl-carnitine, which is converted back to acyl-CoA by carnitine palmitoyltransferase II (CPT II) inside the mitochondria. Within the mitochondria, acyl-CoA undergoes β-oxidation, a cyclic process that sequentially removes two-carbon units to form acetyl-CoA. Each cycle of β-oxidation generates acetyl-CoA, NADH, and FADH_2_, which are crucial for cellular energy production. The acetyl-CoA produced then enters the citric acid cycle. FAO is critical to ensure a continuous supply of acetyl-CoA for the TCA cycle and NADH/FADH_2_ for the electron transport chain, particularly during increased energy demands such as exercise or fasting. The complete oxidation of a 16-carbon fatty acid, such as palmitate, results in the net production of 106 ATP molecules, with three ATP generated per cycle of fatty acid oxidation, followed by additional ATP production through NADH and FADH2 in the electron transport chain and acetyl-CoA entering the TCA cycle.

To summarize, glycolysis converts one glucose molecule into two pyruvate molecules, yielding a net gain of two ATP molecules. It provides intermediates for downstream metabolic pathways essential for cellular homeostasis and stress response. These pyruvate molecules then fuel OXPHOS in the mitochondria, where electron transfer coupled with ATP synthase activity in the respiratory chain generates an additional 30–36 ATP molecules. Its regulation ensures cells adapt to varying energy demands and oxygen availability, maintaining energy homeostasis under diverse physiological conditions (Harris et al., 2012; Carpenter et al., 2015; Martínez-Reyes and Chandel, 2020; Santos, 2021; Lee et al., 2023; Stovell et al., 2023).

Brain cells’ ability to switch their metabolism depending on overall substrate availability and stress conditions underscores the complexity of brain energy metabolism. Alterations of this metabolism after TBI are integral to the brain’s response to trauma. Understanding these responses can help tailor interventions that support cellular energy production, mitigate damage, and promote recovery following traumatic brain injury.

#### 2.1.1 TBI-induced changes in brain bioenergetics

Many human and animal studies have shown that TBI leads to an acute phase of hyper-glycolysis followed by a prolonged phase of metabolic depression (Carpenter et al., 2015; Stovell et al., 2017; Devanney et al., 2020; Xu et al., 2021). In adult patients, this hyper-glycolytic phase lasts several hours to days, and the subsequent hypometabolic phase can persist for weeks, correlating with the severity of the injury and functional deficits (Kansakar et al., 2025). In models of younger animals, the recovery from these metabolic changes is faster than in adults, suggesting age-related differences in the metabolic response to TBI. The acute phase involves increased glucose utilization due to ionic fluxes and neurotransmitter release, while the chronic phase is marked by reduced glucose metabolism, which can be influenced by impaired glycolytic flux and mitochondrial dysfunction (Carpenter et al., 2015; Stovell et al., 2017; Li et al., 2020; Rabinowitz and Enerbäck, 2020; Rose et al., 2020; Xu et al., 2021).

A recent human study (Pinggera et al., 2021b) using Phosphorus-31 Magnetic Resonance Spectroscopy (31P-MRS) imaging in TBI patients highlighted significant alterations in ATP resynthesis, reflected by changes in phosphocreatine (PCr) to ATP ratios (PCr/ATP). Increased PCr/ATP ratios observed in the subacute phase may indicate adaptive responses to energy failure or altered glial activity. Furthermore, decreased inorganic phosphate (Pi) to ATP ratios (Pi/ATP) suggest impaired ATP turnover and hydrolysis, a direct consequence of mitochondrial dysfunction (Pinggera et al., 2021b). However, the devastating nature of brain injury is not restricted to the damaged area with altered bioenergetics, but its spread to healthy surrounding tissue, as shown in two other human studies (Pinggera et al., 2021a; Strope et al., 2022).

While human studies are scarce due to the high expense and lack of tools to perform such analysis, numerous *in vivo* animal studies in both blunt and blast injuries reported devastating alterations to OXPHOS post-injury. A study by Hubbard et al. (2019) demonstrated a significant decrease in mitochondrial respiration 48 h after a single or repetitive closed-head injury in rats suggesting early mitochondrial dysfunction as a key factor in cellular vulnerability to repeated head impacts. Additionally, this group showed that a mitochondrial uncoupler drug, MP201 (Hubbard et al., 2018), significantly improved mitochondrial function, histopathology, and cognitive outcomes (Hubbard et al., 2023). These findings suggest that acute mitochondrial dysfunction can be targeted to provide neuroprotection from reactive oxygen species.

Blast injury also alters mitochondrial function. After blast injury in rats, decreased OXPHOS proteins and increased oxidative stress markers were observed, indicating bioenergetic failure. These findings suggest that targeting mitochondrial function could be a viable therapeutic strategy for blast-induced TBI (Rana et al., 2020; Priemer et al., 2022; Siedhoff et al., 2022; Guilhaume-Correa et al., 2023; Hubbard et al., 2023; Schmitt et al., 2023).

In a rat severe TBI model (penetrating TBI - PTBI), there was a differential response to injury in different brain regions (Pandya et al., 2019; Pandya et al., 2021). Isolated mitochondria from the frontal cortex and striatum rapidly responded, with significant dysregulation detected within 30 min of injury. However, the mitochondrial response resolved to baseline levels before a second, more robust phase of bioenergetic dysregulation was found at 24 h that persisted up to 14 days post-injury. In contrast, mitochondria from the hippocampus, more distal to the lesion, only showed dysfunction starting at 7 days and remaining evident up to 14 days post-PTBI. The results indicated that PTBI-induced mitochondrial dysfunction is time- and region-specific with this severe TBI model. In a rat controlled cortical impact (CCI) model, there was decreased respiration associated with increased oxidative damage to cortical mitochondria. Synaptic mitochondria were more vulnerable than non-synaptic organelles (Hill et al., 2018). Thus, there appear to be significant changes in mitochondrial bioenergetic function after many different types of traumatic brain injury in people and in animal models.

#### 2.1.2 Treatment for bioenergetics

This review focuses on potential bioenergetic biological targets that have not yet been explored for TBI patients. Treatments that are already in clinical trials or FDA-approved are reviewed elsewhere (Ahluwalia et al., 2021). Addressing bioenergetic dysfunction following TBI is a critical component of potential treatments aimed at mitigating neuronal damage and promoting recovery. Several therapeutic approaches target restoring mitochondrial function and overall cellular energy metabolism. The treatment options are summarized in Table 1.

**TABLE 1:** Different potential treatments for mitochondrial dysfunction associated with traumatic brain injury (TBI).

	Treatment	Outcomes
Bioenergetics	Antioxidants (MitoQ and SS-31)	• Decrease ROS ∙ Preserve mitochondrial function and integrity
	Resveratrol and nicotinamide riboside	• Promote mitochondrial biogenesis
	Ketone supplementation	• Improve mitochondrial function ∙ Reduce oxidative stress ∙ Neuroprotective effects
	Cyclosporine A	• Preserve membrane potential ∙ Decrease calcium influx ∙ Prevent release of pro-apoptotic factors
	Methylene blue	• Preserve mitochondrial function ∙ Reduce neuronal apoptosis ∙ Decrease cerebral edema and lesion volume ∙ Decrease ROS ∙ Promote autophagy and inhibits microglial activation
	Intermittent fasting with ketogenesis	• Decrease calcium influx and lipid peroxidation ∙ Elevate antioxidant levels ∙ Improve mitochondrial respiratory complex activity ∙ Improves cognitive function
	Photobiomodulation	• Stimulate cytochrome C oxidase in ETC ∙ Enhance ATP production ∙ Improve membrane potential ∙ Neuroprotective effects ∙ Decrease neuroinflammation and promote neuroplasicity
Metabolism	Glucose	• Prevents hypoglycemia ∙ Improve metabolism ∙ Decrease axonal injury
	Pyruvate	• Increase ATP production ∙ Improve metabolism
	Succinate	• Improve TCA functionality
	Omega-3 fatty acids	• Inhibit neuroinflammation ∙ Decrease necroptosis
Ion homeostasis	Ru360 and minocycline	• Decrease calcium uptake ∙ Suppress production of ROS
	Antioxidants (circumin, vitamin E, resveratrol)	• Neutralize ROS ∙ Lower lipid peroxidation ∙ Modulate inflammatory pathways
Inflammation	Hydrogen sulfide	• Decrease ROS ∙ Reduce production of pro-inflammatory cytokines
	Cytokine C	• Improve lactose clearance ∙ Lower lipid peroxidation ∙ Increase antioxidant activity

A better understanding of the role of mitochondrial dysfunction in the underlying biological events associated with TBI will allow for the development of specific therapeutics for TBI.

One promising strategy involves the use of *mitochondrial-targeted antioxidants*, such as MitoQ and SS-31 (Oyewole and Birch-Machin, 2015; Fields et al., 2023; Du et al., 2024). These compounds specifically accumulate in the mitochondria, where they can effectively neutralize ROS and reduce oxidative stress. They thereby preserve mitochondrial integrity and function by mitigating oxidative damage, ultimately enhancing ATP production and cellular energy balance (Smith and Murphy, 2010; Szeto, 2014). Another drug that targets mitochondria is the mitochondrial uncoupler, MP201, a pro-drug of 2,4-dintrophenol. In a rodent model of mild blast TBI, MP201 improved mitochondrial function and enhanced behavioral recovery (Hubbard et al., 2023).

Another approach focuses on enhancing mitochondrial biogenesis, the process of forming new mitochondria (Jia et al., 2020; Simmons et al., 2020; Vekaria et al., 2020; Jamwal et al., 2021). Agonists of the peroxisome proliferator activated receptor gamma coactivator 1-alpha (PGC-1α) have shown promise in preclinical studies. PGC-1α is a key regulator of mitochondrial biogenesis and oxidative metabolism. Activating this pathway can increase the number of functional mitochondria, thereby improving bioenergetic capacity. Drugs such as resveratrol and nicotinamide riboside have been explored for their potential to activate PGC-1α and promote mitochondrial biogenesis, offering a means to counteract the mitochondrial deficits induced by TBI (Cerutti et al., 2014; Gordon et al., 2018).

*Ketogenic diets and ketone supplementation* have also emerged as potential therapies for bioenergetic dysfunction post-TBI (Eiden et al., 2019; Bernini et al., 2020; Shaito et al., 2020; Har-Even et al., 2021; Thau-Zuchman et al., 2021). These diets are high in fats and low in carbohydrates, producing ketone bodies such as β-hydroxybutyrate. Ketone bodies can serve as an alternative energy substrate for the brain, bypassing the glycolytic pathway and directly entering the citric acid cycle to produce ATP. Preclinical animal studies indicate that ketone bodies may improve mitochondrial function, reduce oxidative stress, and enhance energy metabolism in the injured brain (Eiden et al., 2019; Bernini et al., 2020; Shaito et al., 2020; Har-Even et al., 2021; Thau-Zuchman et al., 2021). Early-phase clinical trials have demonstrated that ketogenic interventions are safe, feasible, and capable of inducing ketosis in patients with acute and subacute TBI (Arora et al., 2022; Edwards et al., 2024). Although current clinical evidence is primarily based on small-scale feasibility and metabolic response studies, they provide preliminary support for the neuroprotective potential observed in preclinical models. Larger randomized controlled trials are needed to validate clinical efficacy, optimize treatment protocols, and establish ketogenic approaches as standard care for supporting the brain’s energy needs during the critical post-injury period.

Intermittent fasting (IF) and calorie restriction represent complementary approaches to ketogenic diets that may offer synergistic benefits for restoring mitochondrial function post-TBI. While animal studies demonstrate that IF enhances mitochondrial biogenesis via cAMP response element-binding protein (CREB) and PGC-1α activation, reduces oxidative stress through Nrf2 (nuclear factor erythroid - 2 related factor 2) pathways, and improves circadian regulation (Anderson et al., 2008; Alirezaei et al., 2010; Fernandez-Marcos and Auwerx, 2011; Moro et al., 2016; Mattson et al., 2018; Sutton et al., 2018; Jamshed et al., 2019; Lettieri-Barbato et al., 2020; Haupt et al., 2021), critical translational gaps remain. While a single 24 h fast post-TBI shows neuroprotection in male rodents, a 48 h fast did not demonstrate a comparable result (Davis et al., 2008), suggesting a need for validating duration-dependent effects in humans, particularly regarding risks of energy deficits during acute recovery phases. Notably, most evidence comes from pre-clinical models using pre-injury IF regimens with limited clinical applicability for accidental TBI (Gudden et al., 2021; Cao et al., 2022; Xu et al., 2022). Studies indicate that active-phase fasting demonstrates superior neuroprotection in animals, though human circadian variations need characterization (Froy et al., 2009; Longo and Panda, 2016; Jeong et al., 2024). While IF improves adenosine monophosphate-activated protein kinase (AMPK)-mediated autophagy and stress resistance, contraindications exist for patients with metabolic disorders or specific nutritional requirements (Bujak et al., 2015; Vasim et al., 2022; Shabkhizan et al., 2023; Eliopoulos et al., 2025). These findings suggest that combining ketogenic diets with circadian-aligned IF protocols may present a mechanistically promising strategy for mitochondrial recovery but requires personalized assessment of an individual’s metabolic status, injury severity, and temporal implementation windows to ensure treatment efficacy and safety.

Methylene blue (MB) represents a promising therapeutic agent for mitochondrial bioenergetic dysfunction post-TBI. MB [3,7-bis (dimethylamino)-phenothiazin-5-ium chloride] is an FDA-approved medication with established safety profiles for treating conditions such as cyanide poisoning and methemoglobinemia (Schirmer et al., 2011). Studies have shown that intravenous administration provides optimal delivery, allowing MB to readily cross the blood-brain barrier and accumulate in brain tissue at concentrations 10–20 times higher than in circulation (Peter et al., 2000). Upon reaching the brain, MB preferentially localizes to neuronal mitochondria, where it serves as a catalytic redox cycler, shuttling electrons directly from NADH to Cytochrome C oxidase (Complex IV) in the ETC. This mechanism effectively bypasses dysfunctional complexes I and III that are commonly impaired post-TBI, thereby potentially restoring electron flow, mitochondrial respiration, and energy metabolism in injured neurons (Rojas et al., 2012). Preclinical studies in TBI models demonstrate that low-dose MB treatments (0.5–5 mg/kg) significantly reduce cerebral edema, attenuate lesion volume, increase neuronal survival, and improve behavioral outcomes with no adverse effects (Talley Watts et al., 2014). In an oxygen-glucose deprivation injury model mimicking TBI conditions, MB treatment inhibited excessive neuronal ROS production, maintained mPTP potential, and increased ATP generation (Lee et al., 2002). Although human clinical trials examining MB for TBI are limited, its established safety profile and promising results in preclinical models warrant further investigation into optimizing treatment timing, delivery methods, and dosing regimens for utmost neuroprotection after TBI injury.

Photobiomodulation therapy (PBMT), using red, near-infrared light, and short-wave infrared light (600–1,870 nm), has emerged as a promising non-invasive therapeutic approach for mitochondrial bioenergetic dysfunction post-TBI, with potential for deeper tissue penetration and enhanced therapeutic outcomes (Golovynskyi et al., 2018; Dos Santos Cardoso et al., 2021; Dos Santos Cardoso et al., 2022; Nairuz et al., 2024). PBMT primarily stimulates Cytochrome C oxidase (Complex IV) in the ETC with specific absorption peaks between 660 and 870 nm wavelengths, facilitating nitric oxide (NO) dissociation and enhancing electron transfer efficiency, oxygen utilization, mitochondrial membrane potential, and ATP production (Wong-Riley et al., 2005; Naeser et al., 2011; Thunshelle and Hamblin, 2016; Hipskind et al., 2018; Gong et al., 2021; Nonarath et al., 2021). When PBMT is applied at appropriate wavelengths, even brief exposures of 60–240 s can boost antioxidant enzyme activity, reducing ROS production and inflammatory processes (Zhang et al., 2014; Silva Macedo et al., 2016; Hamblin, 2017; Heo et al., 2019; Jahani Sherafat et al., 2020). PBMT also modulates mitochondrial dynamics by suppressing fission-related proteins (dynamin-related protein 1, fission 1) while promoting fusion-related proteins (mitofusin 1 and 2), preserving mitochondrial integrity crucial for energy production (Gopalakrishnan et al., 2020; Jeong et al., 2021; Li et al., 2023). Additionally, PBMT activates neuroprotective mitogen-activated protein kinase/extracellular signal-regulated kinase (MAPK/ERK) and phosphatidylinositol 3-kinase/protein kinase B (PI3K/Akt) pathways, which contribute to its anti-inflammatory and anti-apoptotic effects (Bathini et al., 2022; Li and Wang, 2022; Shamloo et al., 2023). Preclinical studies have shown efficacy in TBI models, including reduced edema, improved motor recovery, and lifespan extension in Drosophila (Begum et al., 2015). Clinically, near-infrared wavelengths can penetrate human skull tissues sufficiently to stimulate cortical neurons non-invasively; however, achieving optimal therapeutic efficacy remains challenging due to variability in treatment parameters like wavelength selection, dosing regimens, timing post-injury, pulse frequency modulation, and delivery methods (Barrett and Gonzalez-Lima, 2013; Hamblin, 2016; Henderson, 2024; Lim, 2024). Future directions should include artificial intelligence (AI)-driven protocols and nanoparticle-enhanced delivery (e.g., intracranial/implantable LEDs) to overcome penetration barriers.

Pharmacological agents that *modulate the mitochondrial permeability transition pore (mPTP)* have also shown potential in treating bioenergetic dysfunction post-TBI (Skemiene et al., 2020; Huang et al., 2021; Kent et al., 2021; Yang et al., 2022; Pandya et al., 2023). The mPTP is a key player in mitochondrial dysfunction, and its prolonged opening can lead to cell death. Cyclosporine A and other mPTP inhibitors can prevent the opening of this pore, thereby preserving mitochondrial membrane potential, reducing calcium overload, and preventing the release of pro-apoptotic factors. This approach aims to stabilize mitochondrial function and enhance cell survival following TBI (Xiong et al., 1997; Crompton, 1999).

In conclusion, treatments targeting bioenergetic dysfunction post-TBI focus on reducing oxidative stress, promoting mitochondrial biogenesis, providing alternative energy substrates, and stabilizing mitochondrial membrane integrity. These strategies hold promise in preserving mitochondrial function, enhancing energy production, and improving neurological outcomes in individuals with traumatic brain injury. Continued research and clinical trials are necessary to further validate these approaches and integrate them into comprehensive TBI treatment protocols.

### 2.2 Mitochondrial dysfunction and cellular metabolism

**FIGURE 3:**
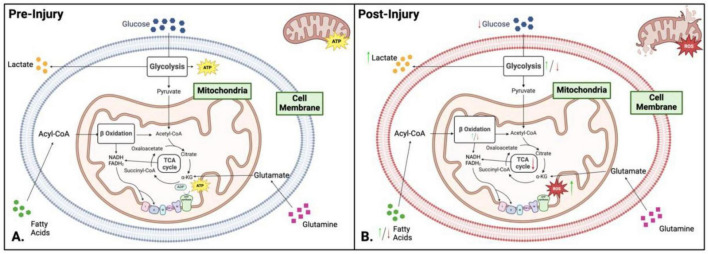
Comparison of metabolism and bioenergetics pre- and post-injury. **(A)** Pre-injury: Under normal conditions, cellular metabolism is well-regulated, with neurons relying on oxidative phosphorylation (OXPHOS) for adenosine triphosphate (ATP) production, while glial cells (microglia and astrocytes) preferentially utilize glycolysis. Key metabolic pathways - including glycolysis, the tricarboxylic acid (TCA) cycle, and OXPHOS – operate efficiently to maintain cellular homeostasis and energy balance. **(B)** Post-injury: Following injury, metabolic activity shifts toward glycolysis as the dominant energy source, enabling rapid ATP production under stress conditions. This metabolic reprogramming results in glucose depletion, intracellular acidosis, increased reactive oxygen species (ROS) and nitric oxide synthase levels, and overall hypo-metabolism. Green and red arrows indicate upregulation or downregulation of specific pathways following injury. Figure created in Biorender.com.

In addition to energy production mitochondria are critical for other cellular homeostasis functions. The ATP generating pathways also produce molecules that are important as building blocks for biomolecule synthesis, as regulators of genomic DNA and as modifiers of post translational modifications. Mitochondria play a central role in cellular metabolism and energy production, making them key mediators in signal propagation for different cellular outcomes (Figure 3A). Mitochondrial dysfunction in metabolism occurs when oxygen and amino acids are available for normal cellular processes, but the mitochondria cannot utilize them effectively due to external trauma (Figure 3B; Amorini et al., 2017; Bhatti et al., 2017). The TCA cycle, glycolysis, and fatty acid oxidation play crucial roles in the biosynthesis of macromolecules and cellular bioenergetics (Figures 2A–C). While most focus on the bioenergetic value of OXPHOS, fatty acid oxidation, or glycolysis pathways, it is vital to explore further the underlying mechanisms of these processes and their link to mitochondrial dysfunction (Figures 3A, B).

Glycolysis and Mitochondrial Dysfunction - An overall decrease in energy production is observed during mitochondrial dysfunction (Rabinowitz and Enerbäck, 2020; Garone et al., 2024). This is due to reduced OXPHOS, ETC malfunction, and increased glycolysis (Figure 3B). Cells will rely on glycolysis for energy production to maintain energy levels during oxidative stress and mitochondria malfunction. While glycolysis is a much faster method of producing energy, it is less efficient and only produces two ATP per glucose molecule. The reliance on glycolysis leads to a high lactate/pyruvate ratio (LPR) due to the reduction of pyruvate into the waste product lactate, which is associated with hypoxia. Continued glycolysis forces the cells to be inflexible in their metabolic processes, leading to continued dysfunction (Carpenter et al., 2015; Jalloh et al., 2017). This hypermetabolism of glucose can lead to hypometabolism in chronic stages, perpetuating mitochondrial dysfunction.

In both mild and severe TBI, two phases occur: hypermetabolism followed by hypometabolism. The initial hypermetabolism state is a response to the influx of glutamate into the synaptic cleft, increasing the amount of ATP needed to activate ion channels. In this state of hypermetabolism, the increased oxygen and glucose demand become too great, and they become depleted (Jalloh et al., 2017; Pandya et al., 2019; Sowers et al., 2021). Once these resources are depleted, the brain switches to anaerobic metabolism, specifically glycolysis, to meet energy demands. This explains why hypoxia is generally associated with increased glycolysis and mitochondria dysfunction in TBI. During this glycolytic state, there is an observed increase in the LPR, leading to acidosis and the breaking down of the cellular membrane, further perpetuating the damaging cycle (Carpenter et al., 2015). Also, a high LPR is associated with poor clinical outcomes in the chronic stages post-injury. Lactate levels are elevated in blast TBI as well (Kumari et al., 2023). High lactate levels also damage the metabolic connection between neurons and glia. Generally, neurons take up astrocytic lactate, which is then converted into pyruvate for the TCA cycle. Increased lactate will accumulate in the extracellular space if this connection is damaged. During chronic stages after TBI, the brain enters a state of hypometabolism and glycolytic depression. In this state, glucose cannot be converted into pyruvate, reducing the amount of acetyl-CoA available for the TCA cycle and leading to depletion of ATP and activation of death pathways.

Fatty Acid Oxidation and Mitochondrial Dysfunction - Fatty acid oxidation (FAO) is a key component of the molecular and metabolic machinery of the brain. FAO is important when glucose levels are low and glycolysis cannot be performed (Szrok-Jurga et al., 2023). Mitochondrial dysfunction affects β-oxidation due to the impairment of the ETC, decreased activity of β-oxidation enzymes, altered metabolite levels, increased reliance on glycolysis, and decreased ATP production (Figure 3B). The NADH and FADH_2_ produced by β-oxidation can accumulate under impaired electron transport chain activity conditions, leading to feedback inhibition of β-oxidation and a significant slowing of the TCA cycle due to high levels of NADH (Martínez-Reyes and Chandel, 2020; Picard and Shirihai, 2022). Along with this, there is an increase in ROS due to the leaking of electrons from the faulty ETC, leading to mitochondrial DNA (mtDNA) damage, inflammation, activation of apoptotic pathways, and further mitochondrial impairment (see section “3 Brain injury-induced secondary mitochondrial dysfunction” for detailed explanation). During mitochondrial dysfunction, there are deficiencies in β-oxidation enzymes like acyl-CoA dehydrogenase, reducing the efficiency of fatty acid oxidation and disrupting metabolic signaling, exacerbating metabolic dysfunction.

Polyunsaturated fatty acids are abundant in brain phospholipids with highly oxidizable structures, making them a target for lipid peroxidation, which is increased due to TBI. However, there is little information on the exact products of lipid peroxidation, their location, or their possible effects. Research shows that both neurotoxic and neuroprotective effects are associated with lipid peroxidation in TBI, so it is critical to understand and identify specific therapeutic targets to develop treatment. After TBI, the accumulation of products from lipid peroxidation contributes to poor clinical outcomes (Anthonymuthu et al., 2017). Also, increases in medium-chain fatty acids, specifically decanoic and octanoic acids, have been associated with poor patient outcomes (Orešič et al., 2016). Decanoic and octanoic acids are also associated with mitochondrial dysfunction by uncoupling metabolic inhibitors of OXPHOS and inducing lipid and protein oxidative damage. Modulations in fatty acid metabolism and astrocytic function occur in blast TBI, as astrocytes attempt to save neurons from oxidative stress by providing non-glucose fuel (Bernini et al., 2020).

Tricarboxylic acid Cycle and ETC - The TCA cycle is the central cellular metabolism and bioenergetics hub (Figure 3A). It is a tightly coordinated series of reactions that drives metabolism within cells (Jalloh et al., 2017; Vakifahmetoglu-Norberg et al., 2017; Martínez-Reyes and Chandel, 2020; Picard and Shirihai, 2022; Garone et al., 2024) (please see the pathway details in see section “2.1 Brain bioenergetics”). Mitochondrial dysfunction significantly impacts the functionality of the TCA cycle (Figure 3B). Damaged mitochondria increase the production of ROS and show metabolite imbalances with altered metabolic flux. Increased levels of ROS damage TCA cycle enzymes and further impair mitochondrial function. Impairment of the ETC results in a backlog of NADH and FADH_2_ produced by the TCA cycle, hampering the progression of the TCA cycle due to the cycle’s reliance on the generation of NAD^+^ and FAD created by the ETC. Alterations in the levels of TCA intermediates result in numerous other sequelae as these intermediates have key functions associated with chromatin modifications, DNA methylation, hypoxic response, and cellular immunity. Metabolism is also shifted from the TCA cycle to glycolysis to meet bioenergetic demands during stress (Martínez-Reyes and Chandel, 2020).

TBI-induced changes in the TCA cycle are not fully understood. Alterations in indicators such as the ATP: ADP and NADH: NAD^+^ ratios after TBI are thought to be related to mitochondrial dysfunction. However, there is little research on the more in-depth details associated with each step of the TCA cycle. Generally post-injury, many have found that Complex I and Complex III of the ETC are damaged, leading to increased ROS and cellular damage (Kansakar et al., 2025). ETC malfunction results in an abundance of NADH from β-oxidation and the formation of α-ketoglutarate (α-KG) shutting down the TCA cycle (Vakifahmetoglu-Norberg et al., 2017; Picard and Shirihai, 2022). This halts the neurons’ main form of energy production. In the hypermetabolic state previously discussed, excess succinyl CoA and acetyl CoA may lead to decreased enzyme function, associated with decreased electron flux. Increased succinate has also been reported in blast TBI, which indicates hypoxia, decreased enzyme activity, and lower ETC functionality (Tretter et al., 2016; Rana et al., 2020).

**FIGURE 4:**
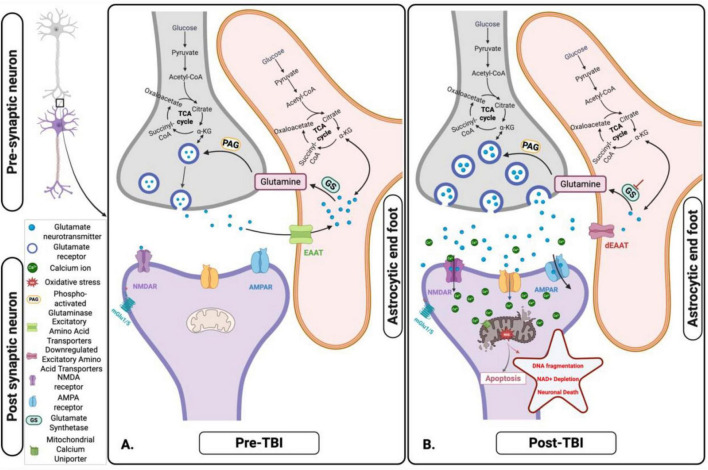
Glutamate-glutamine cycle and its disruption following acute traumatic brain injury (TBI). **(A)** Pre-TBI: Under normal physiological conditions, glutamate released from presynaptic neurons is efficiently taken up by astrocytes via Excitatory Amino Acids Transporters (EAATs). Within astrocytes, glutamate is either converted to glutamine – subsequently shuttled back to neurons for reuse - or metabolized into α-ketoglutarate (α-KG), which enters the tricarboxylic acid (TCA) cycle to support astrocytic energy metabolism and maintain cellular homeostasis. **(B)** Post-TBI: Following TBI, mechanical forces trigger excessive glutamate release and impair astrocytic uptake via EAATs. This dysregulation leads to extracellular glutamate accumulation, resulting in excitotoxicity, mitochondrial dysfunction, and progressive neuronal injury. Figure created in Biorender.com.

**FIGURE 5:**
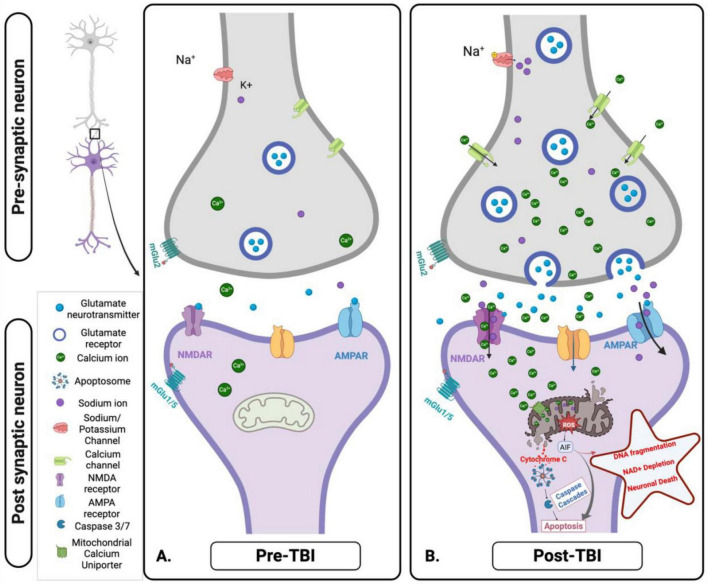
Cellular and mitochondrial ionic dysregulation in response to acute brain injury. **(A)** Pre-traumatic brain injury (TBI): Under normal physiological conditions, ionic homeostasis is maintained at the synapse. Glutamate is released from the presynaptic neuron and binds to N-methyl-D-Aspartate receptor (NMDA) and α- Amino-3-hydroxy-5-methyl-4-isoxazolepropionic acid (AMPA) receptors on the post-synaptic membrane, facilitating the controlled influx of calcium (Ca^2+^) and Sodium (Na^+^). Mitochondria play a key role in maintaining ionic balance and supporting neuronal function through efficient energy production. **(B)** Post-TBI: TBI disrupts this homeostatic balance, causing excessive glutamate release and overactivation of NMDA and AMPA receptors. This results in uncontrolled Na^+^ influx, pathological Ca^2+^ accumulation, mitochondrial dysfunction, ROS production, and initiation of apoptotic signaling pathways. These disturbances contribute to excitotoxicity, oxidative stress, and progressive neuronal injury. Figure created in Biorender.com.

Glutamate excitotoxicity - Glutamate plays a crucial role in mitochondrial dysfunction in TBI (Figures 3–5). Under normal conditions, astrocytes play a central role in regulating glutamate levels in the brain, facilitating neuronal-astrocyte communication to maintain metabolic homeostasis (Figure 4). Glutamate is primarily converted to glutamine by astrocytes and subsequently released into the extracellular space for neuronal uptake and metabolism. Additionally, astrocytes contribute to the TCA cycle by converting glutamate into α-KG, a process essential for energy balance, as it compensates for the ATP expenditure required to maintain sodium and potassium gradients during glutamate uptake (Figure 4A; Sowers et al., 2021). However, TBI disrupts this tightly regulated system, leading to excessive extracellular glutamate accumulation, observed in both mild and severe TBI (Amorini et al., 2017). This dysregulation results in excitotoxicity and metabolic failure, as neurons require increased ATP-dependent ion pump activity to restore ionic equilibrium. The increased mitochondrial demand exacerbates dysfunction, leading to ETC impairments, ROS overproduction, apoptotic signaling, and inflammatory pathway activation (Sowers et al., 2021). In blast-induced TBI, glutamate levels decrease, further compromising metabolism, bioenergetics, and neurotransmission (Rana et al., 2020; Kumari et al., 2023).

#### 2.2.1 Metabolic regulation as treatment post-TBI

Due to the innate complexity of the metabolic machinery and lack of understanding of the biological mechanisms associated with TBI, finding treatments or therapies based on metabolic dysfunction is difficult. However, there have been some promising possible targets that can mitigate the damage associated with secondary injury progression and mitochondrial dysfunction. Some of these treatments are associated with metabolism, including glucose, pyruvate, TCA components (specifically succinate), and Ω-3 fatty acids. Each of these compounds target different sections of metabolism with varying results. The treatment options are summarized in Table 1.

Glucose supplementation has been a controversial topic as there is no clear understanding of glucose metabolism in traumatized brains. Additionally, both hyper and hypoglycemia have a negative effect on the brain post-TBI. In a study where glucose was supplemented to the brain post-TBI, it was found that patients were limited in their ability to metabolize glucose, even when directly delivered into the brain (Stovell et al., 2023). On the other hand, it is important to note that the glucose was delivered into the brain’s extracellular fluid rather than the brain’s natural circulatory system route of obtaining glucose.

Pyruvate has also been suggested as a possible therapeutic for TBI due to its crucial role in metabolism and bioenergetics. Supplementing with pyruvate should push the TCA cycle forward and increase ATP production and metabolism in the hypometabolic state post-injury. Unfortunately, pyruvate supplementation has had mixed results. There were improvements in mitochondrial complex I enzyme but no improvement in motor and sensory performance (Ariyannur et al., 2021).

Succinate’s role in metabolism and ATP production cannot be understated, making it a potential treatment for mitochondrial dysfunction. Direct TCA supplementation of succinate improved metabolism post-TBI in *in vitro* cultures and in humans, thus making it a potential therapeutic for mitochondrial dysfunction in TBI (Giorgi-Coll et al., 2017; Jalloh et al., 2017).

Lastly, supplementation with *Ω-3 fatty acids* has the potential to kick-start the metabolic machinery due to their importance in producing acetyl-CoA for the TCA cycle. Fatty acid supplementation has been found to inhibit neuroinflammation and necroptosis in TBI (Wu et al., 2023) and alleviate neurological impairment, specifically in mice (Zhang et al., 2020). While these are promising results, it is important to remember that successful treatment in mice might not be replicated in people.

## 3 Brain injury-induced secondary mitochondrial dysfunction

Mitochondrial bioenergetic and metabolic dysfunction play critical roles in TBI’s primary and secondary pathophysiology (Figures 5A, B). The disruption of OXPHOS and ATP production within the mitochondria leads to a cascade of detrimental effects, including ionic imbalances, progression of neuroinflammation, and cellular death. Compromised mitochondria fail to produce sufficient ATP to maintain cellular ion gradients, impairing ion pumps like the sodium (Na^+^)/potassium (K^+^)-ATPase, leading to an accumulation of intracellular calcium (Ca^2+^) and Na^+^ ions (Hubbard et al., 2019; Wu et al., 2022). The ionic imbalance caused by mitochondrial dysfunction further exacerbates neuronal damage through Ca^2+^ overload, which disrupts the mitochondrial membrane potential and boosts the production of ROS. The increased ROS levels contribute to oxidative stress, damaging mitochondrial DNA (mtDNA), proteins, and lipids, thus perpetuating mitochondrial dysfunction and cellular injury (Wu et al., 2022). ROS and damaged mtDNA released from dysfunctional mitochondria serve as damage-associated molecular patterns (DAMPs), triggering the activation of microglia and astrocytes, the primary immune cells in the brain. This activation releases pro-inflammatory cytokines and chemokines, propagating a neuroinflammatory response (Peng et al., 2022). At last, damaged mitochondria-released Cytochrome C triggers apoptotic processes by activating caspases (Desagher and Martinou, 2000; Garrido et al., 2006). Apoptotic signaling, oxidative stress, and mtDNA damage collectively exacerbate neuroinflammation and neurodegeneration (Bader and Winklhofer, 2020; McGovern and Barreto, 2021) propagation in the long term (De Gaetano et al., 2021; Hu et al., 2022). The resulting inflammatory environment perpetuates a vicious cycle of mitochondrial dysfunction, oxidative damage, inflammation, and neurodegeneration (Trushina and McMurray, 2007; Fesharaki-Zadeh, 2022; Liaudanskaya et al., 2023; Zhang et al., 2024). The interplay between mitochondrial dysfunction, ionic imbalance, and neuroinflammation highlights secondary injury mechanisms’ complex and interconnected nature following TBI (Hubbard et al., 2019; Peng et al., 2022). The following section will further review the cycle of secondary mitochondrial dysfunction following TBI (Hubbard et al., 2019; Bernini et al., 2020; Wu et al., 2022).

### 3.1 Ionic disbalance as a consequence of bioenergetic failure

The pathophysiological mechanisms underpinning the neuronal damage caused by the interplay between glutamate and ion flux post-traumatic brain injury and mitochondrial bioenergetic and metabolic dysfunction are still poorly understood. Studies suggest that post-TBI, excessive release of glutamate (see section “2.2 Mitochondrial dysfunction and cellular metabolism”), an excitatory neurotransmitter, overstimulates glutamate receptors, such as voltage-gated calcium channels (VGCC) and N-methyl-D-aspartate (NMDA), a-amino-3-hydroxy-5-methyl-isoxazolepropionic acid (AMPA), and Kainate receptors, leading to an influx of Ca^2 +^ and Na^+^ ions into neurons (Stanika et al., 2012; Weber, 2012; Luo et al., 2019; Hoffe and Holahan, 2022). The resultant ionic imbalance and increased intracellular calcium concentration further alter mitochondrial function, creating a vicious cycle of metabolic dysfunction and excitotoxicity, where mitochondrial impairment not only exacerbates glutamate excitotoxicity but is also initially driven by ion flux and glutamate release following TBI (Jeffs et al., 2007; Stanika et al., 2012; Stovell et al., 2017; Granzotto et al., 2020).

A potential mechanism that drives ionic imbalance involves the ionotropic glutamate receptors, specifically the NMDA receptors. After the injury, these receptors cause a massive influx of Ca^2+^ and Na^+^ ions into neurons, setting off a series of events leading to mitochondrial dysfunction and neuronal death (Luo et al., 2019; Figure 5B). The cytosolic Ca^2+^ levels ([Ca^2+^]_*c*_) rise quickly and persistently due to an excessive Ca^2+^ influx via NDMA receptors and VGCC. The mitochondrial calcium uniporter (MCU) absorbs this increased [Ca^2+^]_*c*_, ultimately resulting in mitochondrial Ca^2+^ overload (Cheng et al., 2013).

The elevated levels of mitochondrial Ca^2+^ ([Ca^2+^]_*m*_) disrupt mitochondrial function through several mechanisms. *Mitochondrial permeability transition pore* - elevated [Ca^2+^]_*m*_ can cause the inner mitochondrial membrane’s non-selective channel, the mitochondrial permeability transition pore (mPTP), to open. This allows pro-apoptotic molecules, like Cytochrome C, to be released from the mitochondrial intermembrane space into the cytosol, initiating the intrinsic apoptotic pathway (Springer et al., 2018). *Oxidative stress* - enhancing the electron transport chain’s activity and decoupling oxidative phosphorylation may encourage the generation of ROS. ROS are produced mainly by the mitochondrial respiratory chain, particularly complexes I and III (Cheng et al., 2010). Superoxide anions are created when these complexes experience increased electron leakage. This anion can then be further transformed into hydroxyl radicals and hydrogen peroxide (Pandya et al., 2023). The resultant oxidative stress intensifies mitochondrial dysfunction and brain damage by destroying mitochondrial proteins, lipids, and DNA. *Mitochondrial dynamics* - excessive mitochondrial fission and fragmentation can result from the influx and overload of [Ca^2+^]_*m*_, which can contribute to the disturbance of mitochondrial dynamics. Mitochondria are dynamic organelles that constantly fuse and divide to preserve function and homeostasis and adjust to the cell’s energy requirements. However, in the case of TBI, the equilibrium between fusion and fission is disrupted, which causes the mitochondria to fragment (Cheng et al., 2012; Guilhaume-Correa et al., 2023; Liaudanskaya et al., 2023; Zhang et al., 2024). This fragmentation impairs the production of ATP and facilitates the release of pro-apoptotic factors, further contributing to neuronal dysfunction and death (Figure 5B).

Among these mechanisms, the glutamate/Ca^2+^/ROS axis is a detrimental cycle that prolongs and sustains neuronal injury (Granzotto et al., 2020; Mira et al., 2023). The ROS generated by mitochondria could further induce Ca^2+^ release from the endoplasmic reticulum, partly by modulating the activity of stromal interaction molecule (STIM) proteins. The ROS can oxidize STIM 1 and STIM 2, which act as Ca^2+^ sensors in the endoplasmic reticulum, potentially modifying their ability to regulate store-operated calcium entry (SOCE) (Nelson et al., 2018). STIM oxidation may increase Ca^2+^ influx through the Orai channels, further amplifying the excitotoxic cascade. Additionally, ROS can elevate phospholipase C activity, which produces inositol triphosphate (IP3), triggering more Ca^2+^ release from the endoplasmic reticulum. This process may involve STIM2, which is more sensitive to small changes in the endoplasmic reticulum Ca^2+^ levels than STIM1 (Rao et al., 2015). The positive feedback loop between Ca^2+^ and ROS exacerbates the excitotoxic cascade and mitochondrial dysfunction (Rana et al., 2019; Fesharaki-Zadeh, 2022). ROS can also directly damage cellular membranes, proteins, and DNA, further compromising neuronal viability (Maher et al., 2018). Downregulation of STIM2 has been shown to reduce calcium overload, decrease mitochondrial fragmentation, lower ROS levels, prevent mitochondrial membrane potential loss, and improve ATP synthesis impairment. STIM2 is, therefore, a crucial regulator of SOCE in cortical neurons, and targeting STIM2 could be a viable therapeutic strategy for reducing calcium dysregulation and subsequent mitochondrial dysfunction after TBI (Rao et al., 2015).

#### 3.1.1 Targeting the mitochondrial ion homeostasis post-TBI

Calcium Uniporter: Given the central role [Ca^2+^]_*m*_ overload plays in TBI-induced neuronal injury, targeting the MCU has emerged as a promising treatment strategy. Pharmacological inhibition of the MCU using compounds such as Ru360 and minocycline has shown neuroprotective effects in several human and animal TBI models and models of neurodegenerative diseases (Cheng et al., 2012; Chitturi et al., 2021; Koulaeinejad et al., 2019; Celorrio et al., 2022). These inhibitors lower [Ca^2+^]_*m*_ uptake, suppress the production of ROS, and prevent the mPTP from opening, thereby mitigating neuronal death. Genetic deletion of the MCU can confer neuroprotection in animal models of TBI (Nichols et al., 2018). However, the potential impact of inhibiting MCU on physiological [Ca^2+^]_*m*_ signaling and energy production must be carefully evaluated. Selective inhibition of the MCU only during the acute phase of TBI may provide a therapeutic window for intervention without compromising long-term neuronal function.

Mitochondrial Fission and Fusion: Mitochondrial dynamics encompass fusion and fission processes, which are pertinent in maintaining mitochondrial function and adapting to cellular energy demands. The excessive fission of the mitochondria and fragmentation have been observed to contribute to neuronal dysfunction and death in TBI (Fischer et al., 2016; Di Pietro et al., 2017; Omelchenko et al., 2019; Guilhaume-Correa et al., 2023; Liaudanskaya et al., 2023). Targeting proteins such as dynamin-related protein 1, known to be involved in mitochondrial dynamics, may represent a novel therapeutic approach for mitigating TBI-induced neuronal injury (Qi et al., 2013; Fischer et al., 2016). Mitophagy and other mitochondrial quality control systems are critical in further maintaining cellular homeostasis and eliminating damaged mitochondria. Impaired mitophagy has been implicated in the pathogenesis of many neurodegenerative disorders, including TBI (Zhu et al., 2022; Luan et al., 2023). Therefore, enhancing mitophagy through genetic or pharmacological interventions may promote the elimination of dysfunctional or damaged mitochondria and attenuate neuronal damage in TBI.

Oxidative Stress: Oxidative stress, resulting from the imbalance between ROS production and antioxidant defenses, is another key contributor to TBI-induced neuronal injury. Antioxidant strategies aimed at scavenging ROS and bolstering endogenous antioxidant systems have shown promise in mitigating the deleterious effects of TBI (Fesharaki-Zadeh, 2022). Natural antioxidants such as curcumin, vitamin E, and resveratrol have demonstrated neuroprotective effects in animal models of TBI (Dong et al., 2018; Chen B. et al., 2023). These compounds benefit by neutralizing ROS, attenuating lipid peroxidation, and modulating inflammatory pathways. Also, developing targeted antioxidants, such as the mitochondria-targeted compound MitoQ, has gained significant attention (Zhou et al., 2018). These compounds accumulate specifically within mitochondria and offer improved defense against oxidative damage. In addition to exogenous antioxidants, strategies aimed at enhancing endogenous antioxidant defenses have also shown promise. For instance, the activation of nuclear factor erythroid 2-related factor 2, a master regulator of antioxidant gene expression, can confer neuroprotection in TBI models (Dong et al., 2018). Pharmacological activators of nuclear factor erythroid 2-related factor 2, such as sulforaphane and dimethyl fumarate, may represent novel therapeutic approaches to combat oxidative stress in TBI.

Integrative Approach: The complexity of the excitotoxic cascade and the multifaceted nature of mitochondrial dysfunction in TBI pose significant drawbacks for developing robust and effective therapies. Targeting a single ion channel or pathway may not be sufficient in stopping the progression of neuronal damage. Rather, an integrative and robust approach that combines the modulation of multiple targets, such as NMDA receptors, MCU, voltage-gated calcium channels, and antioxidant systems, may be necessary to achieve optimal neuroprotection. Also, the timing of the intervention is very critical in the context of TBI. While early intervention may be essential to prevent initial excitotoxic damage, sustained treatment may be necessary to tackle or address the progressive and chronic nature of the condition.

### 3.2 Mitochondria dysfunction-induced neuroinflammation

Neuroinflammation occurs after TBI and in neurodegenerative disease. Multiple molecular pathways lead to acute and chronic inflammation in almost all diseases; thus, inflammation has been a pharmacological target for decades. However, TBI clinical trials that completely inhibited inflammation demonstrated detrimental outcomes, indicating that inflammation can benefit the brain’s ability to recover post-trauma (Ramlackhansingh et al., 2011; Mannix and Whalen, 2012; Gyoneva and Ransohoff, 2015; Chiu et al., 2016; Jassam et al., 2017; Clark et al., 2019; Johnson et al., 2023). Thus, understanding the context-dependent mechanisms of inflammation and their effect on brain degeneration or regeneration is crucial for targeted therapy in TBI patients. We will focus here on mitochondrial-induced inflammation in the brain.

**FIGURE 6:**
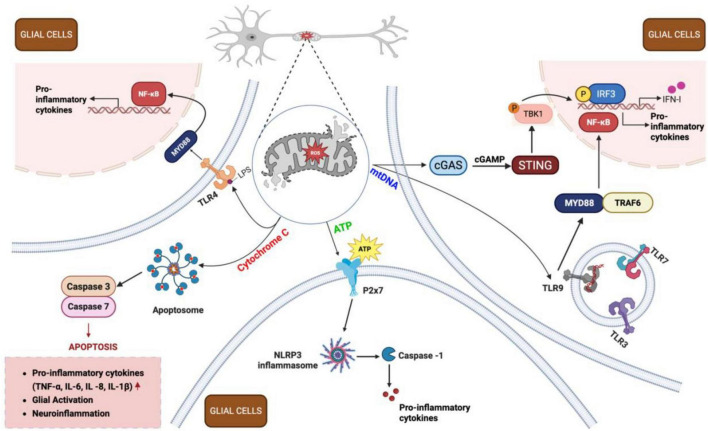
Inflammatory cascade following traumatic brain injury (TBI). Following TBI, damaged neurons release mitochondrial damage-associated molecular patterns (DAMPs) – including adenosine triphosphate (ATP), Cytochrome C, and mitochondrial DNA (mtDNA) - into the extracellular space. These DAMPs activate surrounding glial cells (both microglia and astrocytes) through distinct signaling pathways. ATP binds to purinergic receptors (e.g., P2X7) on microglia, promoting NLRP3 inflammasome formation and cytokine release. Cytochrome C activates TLR4, triggering apoptosome assembly and caspase activation, leading to apoptosis and neuroinflammation. mtDNA stimulates the cGAS-STING pathway in microglia, resulting in IRF3 phosphorylation and IFN-1 production. Additionally, mtDNA engages TLR9 receptors in endosomes, activating nuclear factor kappa B (NF-κB) pathway in both microglia and astrocytes, driving pro-inflammatory cytokine release (IL-1β, IL-6, TNF-α). Together, these processes amplify neuroinflammation through a positive feedback loop, perpetuating mitochondrial dysfunction and sustaining the inflammatory response in injured brain tissue. cGAMP, cyclic guanosine monophosphate–adenosine monophosphate; cGAS, cyclic GMP-AMP synthase; IFN-1, type I interferons; IL, interleukin; IRF3, interferon regulatory factor 3; LPS, lipopolysaccharide; MYD88, myeloid differentiation primary response 88; NLRP3, nucleotide-binding oligomerization domain-like receptor family pyrin domain containing 3; P2x7, purinoreceptor 7; ROS, reactive oxygen species; STING, stimulator of interferon genes; TBK1, TANK-binding kinase 1; TLR, toll-like receptor; TNF-α, tumor necrosis factor alpha; TRAF6, TNF receptor-associated factor 6. Figure created in Biorender.com.

Mitochondrial-induced neuroinflammation post-TBI is a critical aspect of the pathological response, characterized by the release of mtDNA, Cytochrome C, and ATP into the cytosol and extracellular space (Figure 6). These molecules act as DAMPs that activate immune responses, including the STING (stimulator of interferon genes) pathway in microglia, promoting an acute inflammatory phenotype and the release of pro-inflammatory cytokines such as TNF-α and IL-1β. This inflammatory cascade exacerbates neuronal damage and contributes to the chronic phase of TBI pathology.

ATP dysregulation: The central nervous system (CNS) is a key site for purinergic signaling as all brain cell types express purinergic P2 receptors that sense ATP. Extracellular ATP (eATP) maintains CNS homeostasis by modulating microglia, astrocyte, and oligodendrocyte responses. Astrocytic ATP can modulate neuronal excitability, synaptic transmission, and CNS plasticity through exocytotic (vesicular) and non-exocytotic (channels/transporters) mechanisms (Davalos et al., 2005; Cekic and Linden, 2016; Illes et al., 2019). Astrocyte-derived ATP can activate purinergic P2X and P2Y receptors on neurons and other glial cells, depolarizing neurons, increasing neuronal firing, and modulating neurotransmitter release. These effects can be transient or long-lasting, allowing for immediate and sustained modulation of neuronal circuits (Mei et al., 2010; Zarrinmayeh and Territo, 2020). The precise regulation of astrocytic ATP signaling ensures its involvement in physiological processes like neurovascular coupling and synaptic plasticity, as well as pathological conditions such as epilepsy and neuroinflammation (Illes et al., 2019). Microglia, the brain’s resident immune cells, respond rapidly to injury through processes modulated by ATP. The presence of eATP stimulates microglial production of pro-inflammatory cytokines such as TNF-α and IL-1β (Figure 6). Additionally, ATP release can affect astrocytic signaling, influencing neuroinflammatory responses and neuronal survival (Cekic and Linden, 2016; Cao et al., 2018).

During TBI, there is a significant release of ATP, which acts as a danger signal. This release occurs immediately after injury, contributing to the inflammatory response (Moro et al., 2021). For instance, in a controlled cortical impact (CCI) model, a large release of ATP was observed in the cortex and hippocampus, along with increases in glutamate and lactate and decreases in glucose. Blockade of P2Y1 receptors or store-operated calcium channels significantly reduced ATP and glutamate levels post-CCI, indicating the role of these pathways in regulating extracellular ATP (Moro et al., 2021). Overall, ATP plays a dual role in the CNS, participating in both physiological and pathological processes. Its regulation is crucial for maintaining cellular energy balance, modulating inflammation, and ensuring proper neuronal function. Therapeutic strategies targeting ATP signaling pathways hold promise for treating neuroinflammation and other CNS disorders (Davalos et al., 2005; Fiebich et al., 2014; Meyrat and von Ballmoos, 2019; Neupane et al., 2019; Giuliani et al., 2021).

Cytochrome C as a trigger of neuroinflammation: Cytochrome C is an essential protein of the ETC, functioning as an electron shuttle between complex III and complex IV. Under normal conditions, Cytochrome C is in the mitochondrial intermembrane space, playing a vital role in cellular energy production and differentiation. However, during stressed conditions, Cytochrome C can be released into the cytosol, where it activates apoptosis by interacting with apoptosis-protease activating factor 1 and caspases that dismantle the cell (Bertini et al., 2006; Garrido et al., 2006; Wenzel et al., 2019; Figure 6).

Extracellular Cytochrome C (eCytC) significantly affects inflammation and oxidative stress. When released into the extracellular space by damaged astrocytic and microglial cells, Cytochrome C acts as a DAMP, activating immune responses. For instance, eCytC can induce the production of reactive oxygen and nitrogen species by mononuclear phagocytes, enhancing the secretion of cytotoxins. This process is mediated at least partially by toll-like receptor 4 (TLR4) and the JNK signaling pathway, which are crucial for modulating microglial functions (Gouveia et al., 2017; Wenzel et al., 2019; Figure 6). In neuroinflammation, Cytochrome C released from cells undergoing apoptosis can interact with astrocytes, inducing the production of pro-inflammatory cytokines such as IL-1β and IL-8 (Figure 6). Blocking TLR4 with specific antibodies or using TAK 242 can mitigate these effects, highlighting the role of TLR4 in CytC-induced inflammation (Wenzel et al., 2019).

Interestingly, studies have shown that the administration of Cytochrome C can have protective effects in certain conditions. In a model of hemorrhagic shock and reperfusion injury, Cytochrome C administration improved lactate clearance, indicating reduced acidosis. It also decreased hepatic lipid peroxidation, a marker of oxidative stress, and increased the activity of the antioxidant enzyme glutathione peroxidase (Powell et al., 2017). Furthermore, Cytochrome C restored pulmonary levels of the inflammatory cytokine TNF-α to non-injured levels and enhanced the activity of mitochondrial complex I in the liver. These findings suggest that Cytochrome C can limit oxidative stress and inflammation, providing a potential therapeutic approach for oxidative damage and mitochondrial dysfunction (Powell et al., 2017). Moreover, elevated levels of plasma Cytochrome C upon hospital admission in patients with blunt trauma have been correlated with injury severity and survival probability, suggesting its utility as a biomarker for assessing trauma severity and predicting outcomes. Higher plasma Cytochrome C levels were observed in patients with more severe injuries, indicating its potential role in clinical diagnostics and patient management (Eleftheriadis et al., 2016). In summary, Cytochrome C plays a dual role in cellular function and stress response. While it is crucial for electron transport and apoptosis under normal and stressed conditions, its extracellular presence can significantly modulate immune responses and inflammation. Understanding these mechanisms opens up new avenues for therapeutic interventions in diseases characterized by mitochondrial dysfunction and oxidative stress.

mtDNA as a devastating trigger: Mitochondrial dysfunction, often resulting from acute or chronic events such as mutated mtDNA, oxidative stress, drug exposure, compromised mitochondrial dynamics, and aging, plays a significant role in neuroinflammation (Bhat et al., 2015; Bhatti et al., 2017; Fesharaki-Zadeh, 2022; Zong et al., 2024). Cytosolic mtDNA, which refers to the presence of mtDNA in the cytosol, is a major contributor to this inflammatory response. Under normal conditions, DNase prevents an accumulation of cytosolic mtDNA through digestion. However, various mechanisms allow mtDNA to escape into the cytosol or extracellular matrix, including the mPTP, BAX/BAK macropores, and exosome-mediated release, among others (Nicolson, 2014). The opening of the mPTP under stress conditions allows the free movement of small molecules and metabolites into the cytoplasm, including mtDNA, leading to calcium overload, reduced ATP production, and cell death (Elustondo et al., 2016). Similarly, BAX/BAK macropores can form in the mitochondrial membrane, facilitating the release of mtDNA into the cytosol. These events contribute to the neuroinflammatory milieu by activating various immune pathways, including the STING pathway (Decout et al., 2021; Ding et al., 2022; Hu et al., 2022; Kong et al., 2022). Upon detection of cytosolic mtDNA by cGAS (cyclic GMP-AMP synthase), ATP and GTP are converted to cGAMP, which activates the STING pathway, leading to an inflammatory response (McArthur et al., 2018; De Gaetano et al., 2021; Hu et al., 2022; Figure 6).

Traumatic brain injury induces significant mitochondrial dysfunction not only in the brain but also systemically. Raised levels of mtDNA in blood and cerebrospinal fluid after brain injury can be used as biomarkers to determine the severity of injury and the inflammatory cytokine response. Studies have shown that mtDNA can be detected in blood and cerebrospinal fluid post-TBI, providing a means to monitor injury severity and systemic inflammatory responses (Kayhanian et al., 2022). Research using a middle cerebral artery occlusion (MCAO) model in mice showed that the mtDNA-STING axis directs microglial polarization toward a pro-inflammatory phenotype. Inhibition of STING reduced this pro-inflammatory polarization, suggesting that targeting the mtDNA-STING axis could shift microglial polarization toward an anti-inflammatory phenotype, offering potential therapeutic benefits for ischemic stroke and TBI (Kong et al., 2022).

Astrocytes contribute to the inflammatory response post-TBI by releasing mtDNA exosomes. These astrocyte-derived exosomes have been shown to alleviate TBI-induced neuronal defects by reducing oxidative stress and neuronal apoptosis via the activation of Nrf2/HO-1 (heme oxygenase (1) signaling. This mechanism highlights the protective role of astrocyte-derived exosomes in mitigating the effects of TBI (Zhang et al., 2021). Additionally, extracellular vesicles (EVs) containing mtDNA and specific proteins can serve as biomarkers for detecting TBI. A study identified serum amyloid A (SAA) and mtDNA in EVs as novel markers for TBI. Profiling EV content and dynamics through liquid biopsies could revolutionize TBI diagnostics, offering a non-invasive method to assess injury severity and progression (Kumar et al., 2017; Tang et al., 2024). In conclusion, mitochondrial dysfunction and the release of mtDNA play pivotal roles in the neuroinflammatory response following TBI. Understanding the mechanisms of mtDNA release and its impact on immune cell activation opens new avenues for therapeutic interventions and diagnostic tools in managing TBI and related neurological disorders.

#### 3.2.1 Targeting mitochondria-induced neuroinflammation

Anti-inflammatory Strategies: Neuroinflammation plays a significant role in the pathology of TBI and neurodegenerative diseases. However, complete inhibition of inflammation can have adverse effects, as controlled inflammation is necessary for recovery. One promising approach is the use of hydrogen sulfide (H_2_S) donors. H_2_S has been shown to reduce the production of ROS and pro-inflammatory cytokines such as TNF-α and IL-1β in microglia, demonstrating its potential to mitigate neuroinflammation and provide neuroprotection (Kshirsagar et al., 2021; Lin et al., 2024).

Targeting ATP Signaling: Drugs that modulate eATP concentrations in the CNS are emerging as potential therapies for neuroinflammation, given eATP’s roles in synaptic transmission, mood disorders, cortical spreading depression, and microbiota-gut-brain axis signaling (Davalos et al., 2005; Fiebich et al., 2014; Giuliani et al., 2021). eATP is crucial in neuroinflammation and neuronal function. Targeting purinergic receptors (P2X and P2Y), which mediate ATP signaling, can help modulate inflammation and protect neurons. For instance, blocking P2Y1 receptors significantly reduces ATP and glutamate levels, decreasing excitotoxicity and inflammation post-TBI (Engel et al., 2021; Lin et al., 2024). These therapeutic strategies aim to restore ATP homeostasis, reducing neuroinflammatory responses and promoting neuronal recovery.

Modulating Cytochrome C: Cytochrome C release from mitochondria can trigger apoptosis and inflammation. Blocking Toll-like receptor 4 (TLR4) with specific antibodies or using inhibitors like TAK-242 can reduce Cytochrome C-induced inflammation. Additionally, strategies enhancing mitochondrial function and preventing Cytochrome C release, such as antioxidants and mitochondrial stabilizers, can protect against neuroinflammation. These interventions target the pathways activated by eCytC, which acts as a DAMP (Eleftheriadis et al., 2016; Kshirsagar et al., 2021).

Inhibiting the mtDNA-STING Pathway: mtDNA released into the cytosol acts as a danger signal, activating the STING pathway and promoting inflammation. Targeting this pathway offers a therapeutic approach to reduce inflammation. STING inhibitors can reduce pro-inflammatory microglial polarization, as demonstrated in models of ischemic stroke and TBI. This approach can shift the inflammatory response toward a more anti-inflammatory phenotype, potentially improving outcomes (Liao et al., 2020; Decout et al., 2021; Ding et al., 2022; Hu et al., 2022; Ma et al., 2023).

Utilizing Astrocyte-Derived Exosomes: Astrocytes release exosomes containing mtDNA, which can modulate the inflammatory response. These exosomes have been shown to alleviate neuronal defects post-TBI by reducing oxidative stress and apoptosis by activating the Nrf2/HO-1 signaling pathway. Harnessing the protective effects of astrocyte-derived exosomes represents a novel therapeutic strategy to mitigate neuroinflammation and promote neuronal survival (Gharbi et al., 2020; Zhang et al., 2021; Wan et al., 2022; Zong et al., 2024).

Addressing the pathways involved in mitochondrial dysfunction and neuroinflammation post-TBI is crucial for developing effective treatments. By targeting inflammation, ATP signaling, Cytochrome C, and the mtDNA-STING pathway, as well as leveraging astrocyte-derived exosomes, researchers can create combinatorial therapies that reduce neuronal damage and enhance recovery. Continued research into these mechanisms is essential for advancing therapeutic strategies and improving outcomes for patients with TBI and neurodegenerative disorders (Zong et al., 2024).

## 4 Future directions

Addressing mitochondrial dysfunction post-traumatic brain injury requires continued research into several key areas to develop effective treatments and improve patient outcomes. Future research should focus on detailed mechanistic studies to investigate the precise molecular pathways involved in mitochondrial bioenergetic and metabolic dysfunction and their relationship with neuroinflammation and oxidative stress, including the role of mitochondrial permeability transition pores and their inhibitors in mitigating mitochondrial damage and apoptosis. Elucidating the specific contributions of different mitochondrial components such as mtDNA, Cytochrome C, and ATP in triggering and sustaining neuroinflammation is essential.

Targeting mitochondrial mechanisms to develop therapeutics will be critical. Specifically, targeting mitochondrial bioenergetics with PGC-1α agonists, mitochondrial-targeted antioxidants like MitoQ and SS-31, and modulators of mitochondrial biogenesis should yield effective therapies (Table 1). The efficacy of ketone bodies and ketogenic diets in enhancing mitochondrial function and reducing oxidative stress in the injured brain warrants further investigation. The potential of hydrogen sulfide (H_2_S) donors, purinergic receptor blockers, and STING pathway inhibitors in reducing neuroinflammation and promoting neuronal recovery should be explored.

Understanding cell-specific responses is important for comprehending how different cell types (neurons, astrocytes, microglia, oligodendrocytes) respond to mitochondrial dysfunction and contribute to the overall pathology of TBI. It is crucial to analyze the impact of mitochondrial dysfunction on astrocyte-derived exosomes and their role in modulating neuroinflammatory responses and neuronal survival. Characterizing the temporal dynamics of mitochondrial dysfunction across different brain regions and cell types following TBI, including studying the biphasic response in mitochondrial bioenergetics dysfunction in regions like the frontal cortex and striatum compared to the delayed dysfunction in the hippocampus, is vital for understanding long-term effects on neuronal plasticity, cognitive deficits, and neurodegeneration.

Developing biomarkers reflecting mitochondrial status is another promising area for TBI research. EV-containing biomarkers such as mtDNA, Cytochrome C, and serum amyloid A may effectively diagnose and monitor TBI’s severity early. Utilizing advanced imaging and molecular techniques to profile mitochondrial and metabolic changes *in vivo* and correlate them with clinical outcomes is essential for advancing our understanding of mitochondrial dysfunction in TBI. By addressing these future directions and unresolved mechanisms, researchers can develop targeted therapies to mitigate the effects of mitochondrial dysfunction, ultimately improving TBI patients’ recovery and quality of life.

## 5 Conclusion

Mitochondrial dysfunction is a pivotal factor in the pathogenesis of TBI, driving neuroinflammation, oxidative stress, and metabolic disturbances. The release of mitochondrial components such as mtDNA, Cytochrome C, and ATP into the cytosol and extracellular space activates inflammatory pathways, exacerbating neuronal damage and contributing to chronic TBI pathology. Addressing mitochondrial dysfunction is crucial for mitigating secondary brain injury and improving outcomes for TBI patients. Continued research into the underlying mechanisms and therapeutic approaches is essential for advancing clinical treatments for TBI and neurodegenerative diseases.
